# Apelin promotes lymphangiogenesis and lymph node metastasis

**DOI:** 10.18632/oncotarget.2032

**Published:** 2014-05-28

**Authors:** Judit Berta, Mir Alireza Hoda, Viktoria Laszlo, Anita Rozsas, Tamas Garay, Szilvia Torok, Michael Grusch, Walter Berger, Sandor Paku, Ferenc Renyi-Vamos, Bernard Masri, Jozsef Tovari, Marion Groger, Walter Klepetko, Balazs Hegedus, Balazs Dome

**Affiliations:** ^1^ Division of Thoracic Surgery, Department of Surgery, Comprehensive Cancer Center, Medical University of Vienna, Austria; ^2^ Department of Tumor Biology, National Koranyi Institute of Pulmonology, Budapest, Hungary; ^3^ Department of Biological Physics, Eötvös Loránd University, Budapest, Hungary; ^4^ MTA-SE Molecular Oncology Research Group, Hungarian Academy of Sciences, Budapest, Hungary; ^5^ Institute of Cancer Research and Comprehensive Cancer Center, Department of Medicine I, Medical University of Vienna, Austria; ^6^ 1st Department of Pathology and Experimental Cancer Research, Semmelweis University, Budapest, Hungary; ^7^ Thoracic Surgery, National Institute of Oncology and Semmelweis University, Budapest, Hungary; ^8^ Cancer Research Center of Toulouse, INSERM U1037-Université Paul Sabatier Toulouse III, Toulouse, France; ^9^ Department of Experimental Pharmacology, National Institute of Oncology, Budapest, Hungary; Skin and Endothelium Research Division (SERD); ^10^ Department of Dermatology, Medical University of Vienna, Austria; ^11^ Core Facility Imaging, Core Facilities, Medical University of Vienna, Austria; ** These authors share last authorship

**Keywords:** apelin, APJ, lymphangiogenesis, lymph node metastasis

## Abstract

Whereas the role of the G-protein-coupled APJ receptor and its ligand, apelin, in angiogenesis has been well documented, the ability of the apelin/APJ system to induce lymphangiogenesis and lymphatic metastasis has been largely unexplored. To this end, we first show that APJ is expressed in lymphatic endothelial cells (LECs) and, moreover, that it responds to apelin by activating the apelinergic signaling cascade. We find that although apelin treatment does not influence the proliferation of LECs *in vitro*, it enhances their migration, protects them against UV irradiation-induced apoptosis, increases their spheroid numbers in 3D culture, stimulates their *in vitro* capillary-like tube formation and, furthermore, promotes the invasive growth of lymphatic microvessels *in vivo* in the matrigel plug assay. We also demonstrate that apelin overexpression in malignant cells is associated with accelerated *in vivo* tumor growth and with increased intratumoral lymphangiogenesis and lymph node metastasis. These results indicate that apelin induces lymphangiogenesis and, accordingly, plays an important role in lymphatic tumor progression. Our study does not only reveal apelin as a novel lymphangiogenic factor but might also open the door for the development of novel anticancer therapies targeting lymphangiogenesis.

## INTRODUCTION

The lymphatic system is essential for maintaining interstitial fluid homeostasis, immune cell trafficking and lipid transport. The lymphatic network also has an active role in different pathological conditions including tissue inflammation, wound healing, renal and corneal graft rejection [1] but lymphatics are insufficient in patients with primary or secondary lymphoedema [2]. Although lymphatic spread frequently occurs in solid malignancies and is considered an indicator of local dissemination and poor prognosis, a long-established concept has assigned a passive role to lymphatics in tumor progression. However, recent animal studies using lymphangiogenic molecules have indicated that lymphatic vessels interact extensively with malignant cells and, moreover, that lymphangiogenesis is associated with, and can enhance, lymph node (LN) metastasis. The most widely studied molecular system that facilitates the expansion of the lymphatic network both under physiological and pathological conditions is the VEGF-C/-D /VEGFR-3 signaling pathway. Nevertheless, our knowledge of the molecular mechanisms that underlie lymphangiogenesis still lags far behind that of the vascular system [3].

Apelin has been recognized as the endogenous ligand of the human G protein–coupled receptor APJ [4], a member of the seven-transmembrane-receptor family. During embryonic development, APJ expression is largely restricted to the endothelial cells of the developing vascular system [5] and apelin is essential for vascular patterning of the embryo [6]. Nevertheless, apelin and its receptor are strongly expressed in the adult blood vasculature as well [7], and apelin was reported to stimulate blood endothelial cell growth in various *in vitro* [8, 9] and *in vivo* [6, 9] angiogenesis models. It has also been demonstrated that apelin can induce the maturation of tumor blood capillaries [10] and, moreover, that the apelin-APJ system is able to increase the vascularization and growth of different murine tumors [11]. Moreover, apelin was found to be upregulated in some human cancers [12-14], and both our group [15] and others [16, 17] demonstrated a direct association of apelin expression with angiogenesis and/or clinical outcome in malignant disease.

Apelin probably also has lymphangiogenic potential, however, evidence supporting this view has hitherto been obtained only in two very recent studies on the role of apelin in inflammation [18] and lymphatic development [19]. Therefore, and given the apelinergic system's well established angiogenic potential under both physiological and pathological conditions, the present study was conducted to examine additional aspects of the apelin-APJ pathway in lymph vessel growth.

Here, we use *in vitro* and *in vivo* assays to dissect the role of apelin in lymphangiogenesis and lymphatic tumor spread. First, we investigate APJ expressions of LECs and analyze the downstream pathways upon activation of APJ signaling. Next, we assess LECs' *in vitro* proliferation, migration, apoptosis and tube as well as spheroid formation following apelin treatment. We also quantify the *in vivo* effect of exogenous apelin on lymph vessel growth in the Matrigel plug system. Finally, we study whether transfection of tumor cells with apelin expression constructs results in an increase in lymphangiogenesis and LN metastasis *in vivo*. Our results provide the first direct evidence that apelin induces lymphangiogenesis and lymphatic metastasis.

## RESULTS

### APJ is expressed by human LECs and apelin activates its downstream signaling

**Figure 1:**
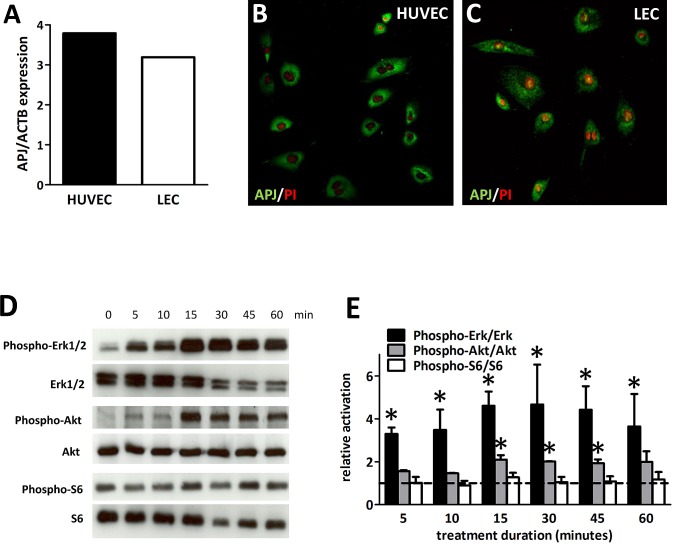
Expression and activation of APJ receptor in LECs (A) In LECs, APJ mRNA was detected at a comparable level to that in HUVECs. (B), Immunofluorescent staining of APJ (*green*) in HUVECs and LECs. Nuclei were labeled with PI (*red*). (D), Time course of phosphorylation of Erk1/2, Akt and S6 following 1 μM apelin-13 treatment of LECs. (E), Quantification of activation by the ratio of the phosphorylated and total protein levels shows robust and significant activation of Erk1/2 and Akt. No significant activation of S6 was found upon apelin treatment. *Asterisks* designate significant differences (P<0.05). Columns represent mean of three experiments; bars, SEM.

RT-PCR and immunocytochemical analysis of HUVECs(human umbilical vein endothelial cells) and LECs demonstrated the presence of APJ mRNA (Fig. 1.A) and protein (Fig. 1. B-C) expressions, respectively. In order to demonstrate that APJ is functionally active in LECs, we investigated the activation of previously described downstream signaling molecules [8, 20]. We found that apelin led to a significantly increased phosphorylation of Erk1/2 and Akt after 5 and 15 minutes treatment, respectively. Activation of S6 was not significant following apelin treatment (Fig. 1. D-E). Thus, APJ expressed in human LECs responds to apelin treatment by inducing the activation of the Erk and PI3K-Akt pathways as we described in other cell types as well [8, 20].

### Apelin does not increase the *in vitro* growth of LECs but promotes their migration and protects them against apoptosis

**Figure 2:**
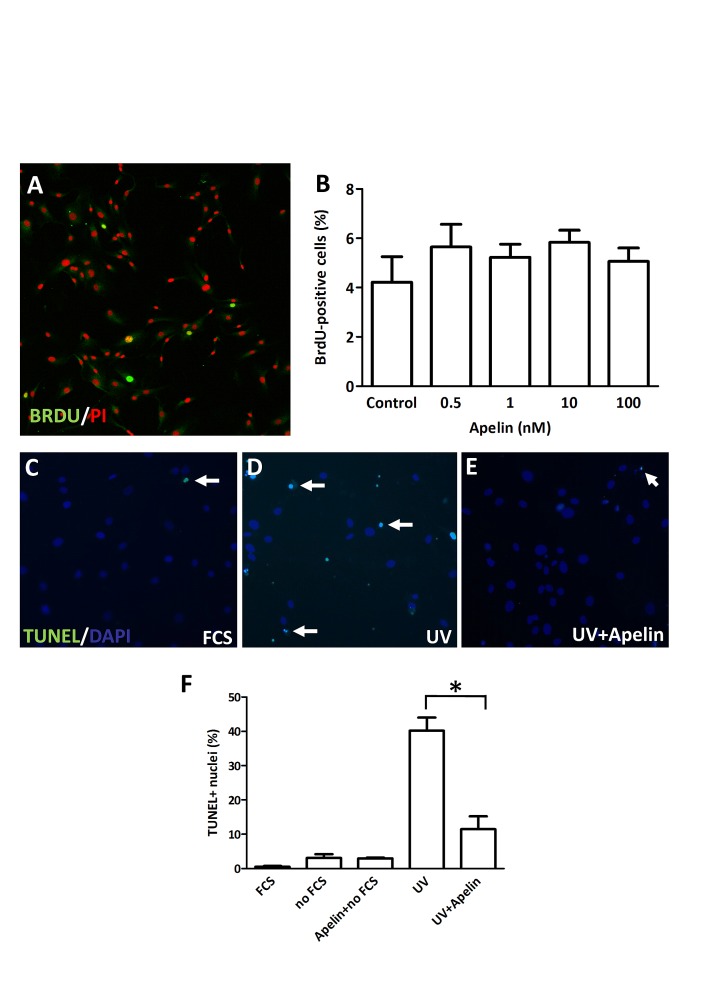
Proliferation and apoptosis of LECs following apelin treatment (A), LECs were cultured in serum-free medium and treated with apelin. After 96 hours, cells were pulsed with BrdU, fixed and stained with an anti-BrdU antibody (*green*) and PI (*red*). (B), No significant change was found in the ratio of BrdU-labeled nuclei upon apelin treatments. (C-E), Apoptosis induction with UV in LECs. Apoptotic cells are labeled with TUNEL (*green*). DAPI nuclei staining is *blue*. *White arrows* indicate apoptotic nuclei. (F), Robust apoptosis was induced by UV irradiation. Apelin treatment significantly reduced the ratio of UV induced apoptotic cells. *Asterisks* designate significant differences (P<0.05). In (B) and (F), columns represent mean of three experiments; bars, SD.

To investigate whether apelin alters LEC growth *in vitro*, the effect of apelin treatment on cell proliferation was studied by BrdU incorporation assay. Of note, exogenous apelin did not alter the *in vitro* proliferation rate of these cells, when compared with untreated cells after 96 h (Fig. 2. A-B).

Apelin has been shown to reduce apoptosis in various cell types, including retinal Müller cells [21], osteoblasts [22], neurons [23], vascular smooth muscle cells [24] and blood endothelial cells as well [6]. We used TUNEL analysis to investigate whether the apelin-APJ system can also inhibit programmed cell death in LECs. Using a standard serum starvation protocol, we found that 48 h serum deprivation did not result in significant apoptosis induction in LECs. However, administration of 1 μM apelin significantly suppressed UV irradiation-induced apoptosis of LECs (P<0.05; Fig. 2. C-F).

**Figure 3:**
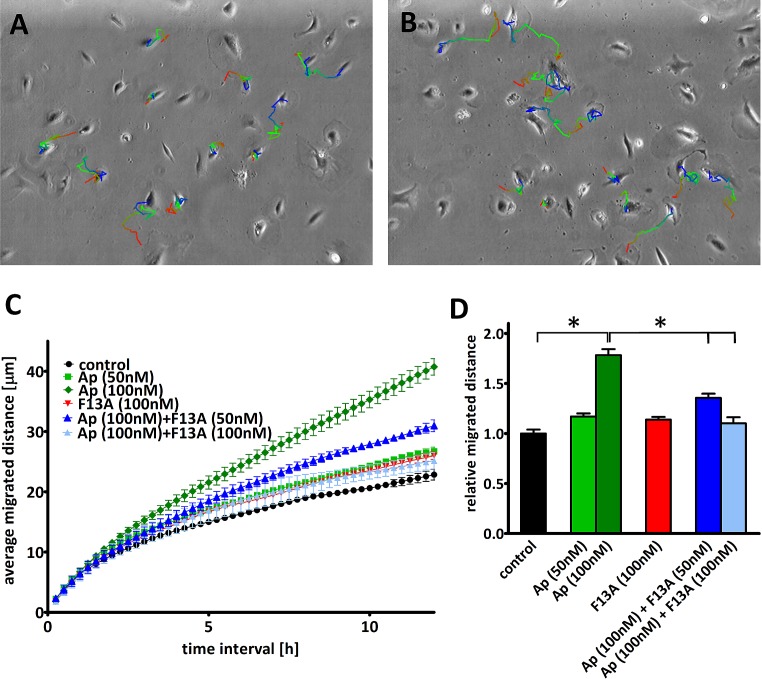
Effect of apelin on LEC migration (A-B), Representative trajectories on the last phase contrast image of the path of LECs with (B) or without (A) apelin treatment. The color of the depicted trajectories refers to the elapsed time in the order from red–green–blue. (C), Average migrated distances of human LECs after incubation with apelin-13 and/or F13A (an APJ antagonist). (D), 12h average migrated distance of LECs after incubation with 100nM apelin-13 significantly increased cell migration and it was reduced in a dose dependent manner by the addition of F13A. *Asterisks* designate significant differences (P<0.05).

Next, we sought to determine the effect of apelin on human LEC migration. Using long-term time-lapse videomicroscopy in 2D cell cultures [25] (Fig. 3. A-B), we found that apelin at 100 nM significantly increased cell migration (P<0.05; Fig. 3. C-D), indicating a pro-migratory potential for apelin in addition to its anti-apoptotic role in human LECs. Importantly, while APJ inhibition by F13A (an APJ antagonist) alone did not result in significant changes in cell migration, it reduced apelin-induced LEC migration in a dose-dependent manner with complete inhibition when apelin and F13A was given at 1:1 ratio (P<0.05; Fig. 3. C-D). Representative pictures of the LECs' trajectories and their average migrated distance for 12 h intervals are also shown in Figure 3. A-B.

### Apelin increases LEC spheroid numbers and stimulates capillary-like cord formation of LECs *in vitro* and, moreover, promotes the growth of lymph vessels in the Matrigel plug model *in vivo*

**Figure 4:**
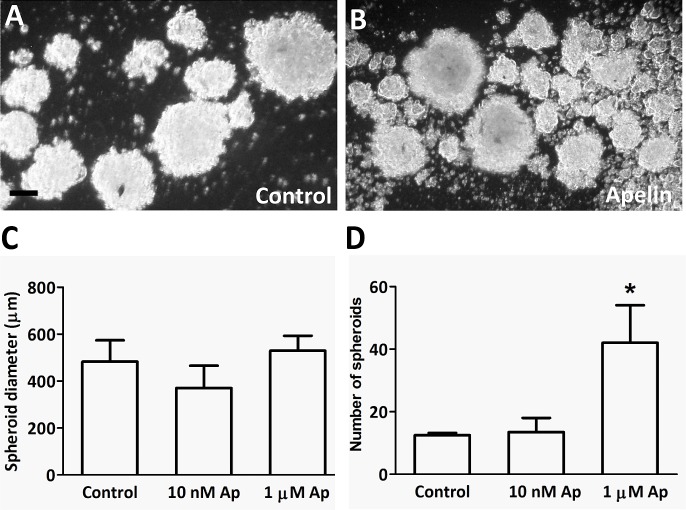
LEC spheroid formation in the presence of apelin (A-B), LECs were plated in serum-free medium in non-adherent plates with apelin-13. After 96h incubation, all spheroids were photographed. (C-D), 1μM apelin-13 significantly increased the number of spheres without affecting their average diameter. Columns represent mean of three experiments; bars, SD; *, P<0.05 vs. control. Scale bar 100 μm

Three-dimensional spheroid endothelial cell models (introduced by Korff and Augustin in 1998 [26]) are useful tools for the analysis of the activity of pro- and anti-angiogenic factors. Because apelin-APJ signaling has been reported to enhance proliferation and assembly of blood endothelial cells in spheroid cultures [27], we also tested the effects of apelin on the formation of multicellular spheroid structures by LECs (Fig. 4). Although apelin treatments at different doses (10 nM or 1 μM) did not result in significantly elevated mean spheroid diameters (Fig. 4. C), apelin (at 1 μM) significantly increased the number of LEC spheroids, when compared with untreated cells after 96 h (P<0.05; Fig. 4. D).

**Figure 5:**
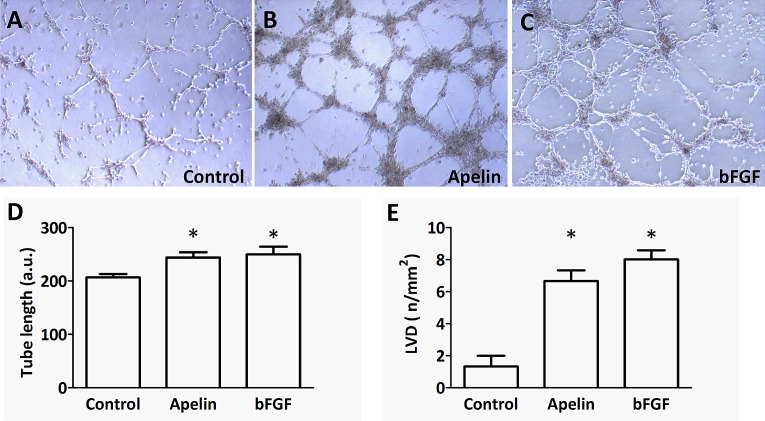
Tube formation capacity of LECs upon apelin treatment *in vitro* and *in vivo.* (A-C), LECs (10^5^) were plated on growth factor-reduced Matrigel-coated tissue culture plates in serum-free endothelial culture medium. 1 μM apelin-13 or 20 ng/ml bFGF was added to the medium. Capillary-like structures within the Matrigel layer were photographed after 18 hours. (D), Both apelin and bFGF significantly increased the average length of tubes (*, P<0.05 vs. control). Columns represent mean of three experiments; bars, SD; a.u., arbitrary unit (E), Matrigel containing PBS, apelin-13 (0.25 μg) or bFGF (0.25 μg) was injected subcutanously into mice. Both bFGF and apelin-13 significantly increased the density of LYVE-1 stained tubular structures in the sections of one-week old plugs. Columns represent means of three experiments; bars, SD; *, P<0.05 vs. control. LVDs are mean lymph vessel counts per square millimeter.

Endothelial tube formation assays are also regarded as *in vitro* models of more advanced stages of (lymph)angiogenesis and are also often used to test the effects of different pro- and anti-(lymph)angiogenic molecules [28]. Thus, we examined the ability of apelin to promote LEC tube formation in a Matrigel tube formation assay and found that apelin (at 1 μM) was effective in promoting LEC differentiation into vascular structures *in vitro* (P<0.05 vs. control; Fig. 5. D). The extent of this effect was comparable to that of bFGF (basic fibroblast growth factor) at 20 ng/mL, which was used as a positive control (P<0.05 vs. control; Fig. 5. D).

The Matrigel plug technique was used to study the effect of apelin on the invasive growth of lymphatic capillaries in mice. Corroborating the lymphangiogenic potential of apelin seen in the above described *in vitro* assays, LVDs were significantly higher in Matrigel plugs containing apelin (0.25 μg) or bFGF (0.25 μg) than in those with PBS only (P<0.05 in case of both apelin and bFGF); Fig. 5. E).

### Apelin overexpression confers a growth advantage to tumor grafts, induces intratumoral lymphangiogenesis and promotes lymphatic metastasis

**Figure 6:**
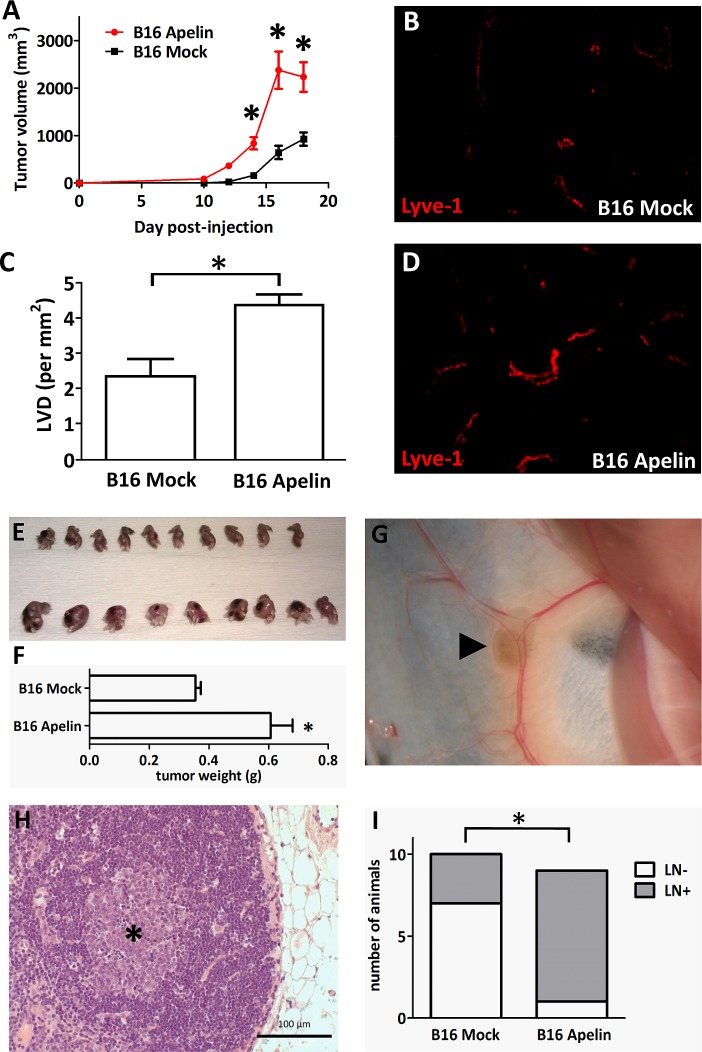
*In vivo* growth of apelin expressing tumor cells (A), Overexpression of apelin through genetic manipulation significantly stimulated the *in vivo* growth of murine B16 melanoma cells in C57BL/6 mice. Growth curves of control vector (B16 Mock)- and apelin-transfected (B16 Apelin) cells. • and ■, means for 10 mice per group; bars, SD; *, P<0.05, versus control. (B-D), Apelin overexpression increases tumor-induced lymphangiogenesis *in vivo*. Frozen sections of 18-day-old control (B) and apelin-overexpressing (D) tumors were stained for the LEC marker LYVE-1 (*red*). Magnification, x200 (B, D). (C), LVDs (mean lymph vessel counts per square millimeter) of 18-day-old apelin-overexpressing or of control B16 tumors. Columns, means for ten mice per group; bars, SD; *, P<0.05 vs. control. (E-F), Apelin-overexpressing B16 cells also developed significantly larger tumors (lower row) when injected into the footpads of mice (*, P<0.05). Ipsilateral inguinal LN regions were carefully examined under a stereomicroscope, photographed and removed (G) (*arrowhead* shows pigmented B16 cells in the LN). (H), Histological demonstration of LN metastasis of B16 melanoma. *Asterisk* shows the colony of metastatic B16 cells in a LN. (I), The percentages of metastatic ipsilateral inguinal LNs (as evaluated by stereomicroscopy and histology) were 89% and 30% in the B16-Apelin and B16-Mock groups, respectively (*, P<0.05).

We were also interested in investigating whether transfection of tumor cells with apelin expression constructs results in an increase in tumor lymphangiogenesis *in vivo*. Our studies to address this goal involved experiments with B16 mouse melanoma cells stably transfected with murine apelin (B16-apelin) or with an empty plasmid vector (B16-mock) [29]. In the first set of animal experiments, we implanted tumor cells subcutaneously into the upper back of mice and assessed tumor volumes (Fig. 6. A) and intratumoral LVDs (Fig. 6. B-D). Importantly, morphometrical analysis using LYVE-1 as a LEC marker (Fig. 6. B, D) revealed significantly higher LVDs present in the apelin-overexpressing tumors as compared with controls (P<0.05; Fig. 6. C). In addition, as expected [29], tumor growth was significantly accelerated in mice injected with B16-apelin cells compared with mice carrying B16-mock tumors (Fig. 6. A).

Stimulation of intratumoral lymph capillary formation by apelin raised the possibility that this peptide might also enhance lymphatic metastasis. Thus, to further explore the role of apelin in lymphatic tumor progression, B16-apelin and B16-mock cells were also injected into the right hind footpads of mice in groups of ten animals per cell line. Two weeks after implantation, B16 tumors increased rapidly in size. Three weeks after injection, when the primary tumors were 5–9 mm in diameter (Fig. 6. E-F), the animals were anaesthetized, and the ipsilateral inguinal LN regions were carefully examined under a stereomicroscope. In case of each animal, the LNs as well as the surrounding fat tissues were then removed and embedded into paraffin for serial sectioning. 8 of 9 (one mouse died at day 2 after tumor implantation in this group) animals (89%) in the B16-apelin group showed metastases by stereomicroscopic inspection (Fig. 6. G; metastatic LN from one representative mouse) and histology (Fig. 6. H). By contrast, only 3 of 10 animals (30%) in the B16-mock group had LN metastases (Fig. 6. I; P<0.05).

## DISCUSSION

Major progress has been made over the past two decades in understanding the molecular regulation of lymph vessel formation. Recent studies on lymphangiogenesis have been focused on two molecules of the VEGF (vascular endothelial growth factor) family, VEGF-C and -D, which are the ligands for VEGFR-3 (VEGF receptor-3) [1, 3]. Although various therapeutic drugs have already been generated to target this molecular machinery, recent results suggest that lymphangiogenesis is a complex process which is regulated by additional signaling pathways [3, 30]. Further efforts are, therefore, needed for the better understanding of lymphatic biology to identify novel lymphangiogenic molecules and to develop more effective treatments for lymphangiogenesis-related diseases, including cancer. In the present study, we provide evidence that apelin acts both *in vitro* and *in vivo* as a lymphangiogenic factor and, accordingly, that it has an important role in LN metastasis.

First, we showed that LECs express APJ *in vitro* and that apelin acts through APJ to increase Erk and Akt phosphorylation. In a recent study investigating the functions of APJ signaling during lymphatic development in zebrafish embryogenesis, Kim et al. also found that the knockdown of APJ in LECs selectively reduced the basal levels of phospho-Akt, but without affecting the phosphorylation status of Erk [19]. These authors, however, investigated the impact of APJ signaling abrogation on the basal phospho-Erk levels of LECs in the presence of FBS (fetal bovine serum), while we analyzed the effect of apelin stimulation in serum-deprived LECs, which raises the possibility that the lack of decrease in Erk phosphorylation in their study was due to their different experimental approach. The results presented in the current paper are indeed consistent with our group's earlier studies reporting that apelin induces the activation of both the PI3K-Akt and the Erk intracellular cascades in HUVECs (with endogenous APJ expression) and also in Chinese hamster ovary cells that have been stably transfected with APJ [8, 20]. Of note, both Akt [22, 24] and Erk [31-33] have also been shown to be downstream effectors of APJ signaling in various additional cell types including osteoblasts, vascular smooth muscle cells, embryonic kidney cells, neurons and cardiomyocytes (reviewed in ref. [34]). Additionally, VEGF-C/VEGFR3 in LECs are also known to activate Akt and Erk signaling and, moreover, both molecular pathways have been implicated in both developmental and pathological lymphangiogenesis [35, 36]. Therefore, further studies are required to better understand the complex crosstalk among these intracellular cascades during lymphangiogenesis.

APJ activation has been demonstrated to promote cell migration in a variety of cell types including retinal endothelial [9], Müller glial [37], oral squamous cell carcinoma [16] and mesendodermal [38] cells. In line with these studies, we also found a significant pro-migratory effect of apelin on LECs. Of note, our results gained by using long-term time-lapse videomicroscopy in 2D cell cultures are also in accordance with the findings of the recent study of Sawane et al. [18], in which they used a transwell migration assay to evaluate the migratory response of LECs to apelin. It is also important to mention that the pro-migratory effect of apelin was reduced in a dose-dependent manner by the addition of F13A (an apelin mutant that has been considered as an APJ antagonist [14, 37, 39]). Our current findings, thus, suggest that F13A acts as a competitive antagonist on LECs when apelin-13 is present.

EC spheroids are increasingly used for evaluating the pro- and anti-angiogenic potential of various factors and drugs [40]. Furthermore, it has been demonstrated that endothelial cells retain differentiated phenotypic properties in 3D spheroids while they tend to lose them in monolayer cultures [26]. Accordingly, we also evaluated the effects of apelin on the formation of LEC spheroids, and found that although it did not have an effect on spheroid sizes, it increased significantly their numbers. This observation, taken together with our further *in vitro* finding that apelin stimulates the capillary-like tube formation of LECs, suggests an important role for apelin in the development of patent lymph capillaries via promoting a pro-adhesive state in the lymphatic endothelium. This assumption is also supported by recent studies reporting that apelin induces the endothelial expression of various intercellular adhesion molecules [10, 41, 42] and, accordingly, that it enhances the integrity of both blood and lymph capillaries [18, 43].

The apelinergic system is expressed in a diverse range of tissues with particular predominance in the blood vasculature [34, 44]. Accordingly, apelin has been shown to stimulate blood vascular EC growth in various *in vitro* [8, 9] and *in vivo* [6, 9] angiogenesis systems, including a mouse model of cancer [11]. Earlier studies have also provided evidence for apelin expression in human glioblastoma [12] and breast cancer [13]. We demonstrated recently a direct association of apelin expression with angiogenesis and clinical outcome in human non-small cell lung cancer [15] and, moreover, showed that autocrine apelin/APJ signaling participates in human colorectal cancer growth [14]. Another group demonstrated the prognostic significance of apelin expression in oral squamous cell carcinoma as well [16]. Most recently, circulating apelin levels were reported to be significantly increased in cancer patients (vs. healthy controls) and, moreover, to correlate with disease stage and prognosis [17]. Nevertheless, although the role of the apelinergic system during tumor progression and blood vessel formation has been an emerging focus of cancer and vascular research in recent years, whether and how apelin/APJ signaling contributes to tumor-induced lymphangiogenesis and lymphatic metastasis remains unclear. Thus, given the biological and clinical significance of apelin in the progression of human cancers, we hypothesized that it might enhance tumor progression by exerting a stimulatory effect on lymph vessel formation as well. Investigating the lymphangiogenic activity of apelin in the *in vivo* Matrigel plug model, we found that apelin-containing Matrigel plugs had significantly higher lymph vessel counts than those with PBS alone. This effect of apelin was comparable to that of bFGF which is known as a potent lymphangiogenic factor [45]. More importantly, apelin overexpression of murine B16 melanoma cells significantly increased the burden and intratumoral lymphangiogenesis of tumors growing subcutaneously in mice. These data were corroborated by the observation of enhanced LN metastasis in mice carrying apelin-transfected primary tumors. Two recent studies from Sawane et al. reported that apelin decreases UVB-induced lymphatic capillary hyperpermeability [18] and stabilizes lymph capillary walls in a high-fat diet mouse model [43]. These data further support our results and, taken together with our findings, suggest that the apelinergic system regulates the adult lymphatic endothelium under both physiological and pathological conditions. The very recent paper of Kim et al. about the essential role of the apelin/APJ pathway during embryonic lymphatic development [19] puts this idea into an even broader perspective implying that the effects of apelin/APJ signaling on LECs are conserved from fishes to mammals and throughout embryonic development to adulthood.

In conclusion, our study identifies apelin as a novel tumor lymphangiogenic factor that enhances LN metastasis. Accordingly, and based also on our group's previous studies, the apelinergic system stimulates tumor progression through the following mechanisms: 1./promotion of blood capillary formation [11, 15], 2./autocrine growth stimulation of malignant cells [14], and 3./enhancement of intratumoral lymphangiogenesis and LN metastasis. Development of potential future therapies targeting this complex pro-tumor function of the apelin/APJ pathway is thus especially intriguing as it might offer benefits to patients with malignant diseases.

## MATERIALS AND METHODS

### Cells

The B16 mouse melanoma cells stably transfected with murine apelin (B16-apelin) or with the empty plasmid vector (B16-mock) have been described in ref. [29]. HUVECs were purchased from Lonza (Walkersville, USA) and cultured in the manufacturer's recommended medium (EGM^®^-2 BulletKit^®^, Lonza) at 37°C in a humified incubator with 5% CO_2_. Telomerase reverse transcriptase (TERT)-transduced LECs (isolated from human foreskins, and generated and characterized as described previously [46, 47]), were grown in the same environment but in the EGM^®^-2-MV BulletKit^®^ (Lonza) medium.

### RNA isolation and real-time PCR

Total RNA was extracted from HUVECs and LECs using TRIzol® Reagent® (Invitrogen) and purified with DNAfree DNase kit (Applied Biosystems, California, USA) according to the manufacturer's protocol. 2 μg of total RNA from each sample were reverse transcribed using High-Capacity cDNA Reverse Transcription kit (Applied Biosystems) according to the manufacturer's protocol. Quantitative real-time PCR was performed using the cDNA as template with TaqMan Universal PCR Master mix (Applied Biosystems) and TaqMan premade gene expression assays (Applied Biosystems) to amplify APJ (Hs00270873_s1). All reactions were conducted as follows: 50ºC for 2 min and 40 cycles of 10s at 95ºC and 1 min at 60ºC in an Applied Biosystems 7500 Real-time PCR System. All samples were assayed in triplicate and control water samples were included in each experiment. The endogenous expression reference was the β-actin gene (Hs03023880_g1).

### APJ receptor protein expression in HUVECs and human LECs

For immuncytochemical stainings, HUVECs and human LECs (10^5^ cells/well of a 24-well plate) were plated on fibronectin-coated coverslips. After 16 hours, cells were fixed in 4% PFA (10 min) and permeabilized with 0.1% Triton-X 100 solution (Sigma-Aldrich Co.) for 1 minute. Then the cells were incubated with a mouse anti-human APJ antibody (1:20; R&D Systems, Minneapolis, USA). Biotinylated anti-mouse IgG (Vector Laboratories, California, USA) served as the secondary antibody and was detected by fluorescein streptavidin (Jackson Immunoresearch, West Grove, USA). For the negative control, the primary antibody was omitted and only the secondary antibody was applied. Finally, counterstaining was performed using propidium-iodide (PI; Partec, Görlitz, Germany). All samples were viewed by confocal laser scanning microscopy using the LSM 700 system (Carl Zeiss AG, Jena, Germany).

### *In vitro* Cell Proliferation Studies

Cell proliferation was examined using the 5-bromo-2'-deoxyuridine (BrdU; Sigma-Aldrich Co.) incorporation assay. 4×10^3^ human LECs per well were plated in a flat-bottomed 96-well plate in serum-free medium, and were treated with apelin-13 (Phoenix Pharmaceuticals, Karlsruhe, Germany) at 0.5, 1, 10 and 100 nM concentrations. After 96 h, 2 mg/ml BrdU was added into the medium and the cells were incubated for an additional 2 hours at 37°C. BrdU-positive cells were labeled with anti-BrdU antibody (Becton-Dickinson, New Jersey, USA). Biotinylated anti-mouse IgG (Vector Laboratories) served as the secondary antibody and was detected by fluorescein streptavidin (Jackson Immunoresearch). Finally, all nuclei were stained with PI (Partec). Three random fields from 3 wells were photographed and the number of BrdU-positive and PI-positive cells in each field counted. The percentage of the BrdU-positive cells was calculated and averaged.

### LEC spheroid growth assay

To establish spheroid cultures from LECs, 3×10^4^ cells were seeded in triplicate in DMEM/Ham's F-12 medium (PAA Laboratories, Pasching, Austria) supplemented with 20 ng/ml bFGF; (Eubio, Wien, Austria), 20 ng/ml epidermal growth factor (EGF; Sigma) and 2% B27 supplement (PAA Laboratories) in ultra low attachment 24-well plates (Corning Inc., New York, USA). Apelin-13 was added to the cells at 10 nM or 1 μM at the time of seeding. 96 hours after plating all spheroids in each well were photographed. In order to exclude small cell clumps from the analysis, only the spheres with a diameter over 100μm were counted and their diameter measured on the digital photographs using Image-J software.

### Migration assay

The motility responses of human LECs to apelin were analysed by long-term time-lapse videomicroscopy in 2D cell cultures as described recently [25]. Briefly, LECs were plated in a 24-well plates (Corning Inc.) in a serum-free EBM-2 medium. The medium was changed to CO_2_-independent medium (Gibco, Paisley, UK) after the overnight cell attachment and apelin-13 or apelin-13(F13A) (an APJ antagonist) [14, 37, 39] was added into the medium at 50 or 100 nM concentrations. Cells were kept in a custom designed incubator built around an inverted phase-contrast microscope (World Precision Instruments, Florida, USA) at 37°C and room ambient atmosphere. Images of 3 neighbouring microscopic fields were taken in every 5 min for 1 day before and 2 days after the treatment. For migration data, pictures were analysed individually with a cell-tracking program enabling manual marking of individual cells and recording their position parameters. The parameter migrated distance is calculated by averaging for each cell the displacement for the first 24 hour interval after treatment, in two independent experiments and 4 microscopic fields.

### Tube formation assay

Liquefied 10 mg/ml Matrigel Basement Membrane Matrix (0.289 ml/well; BD Biosciences, New Jersey, USA) was used to coat wells of a 24-well plate and then allowed to polymerize at 37°C for a minimum of 30 min. 10^5^ cells per well were seeded on basal membrane extract in serum-free, EBM-2 medium (Lonza). The cells were treated with 1 μM apelin-13 (Phoenix) or 20 ng/ml FGF (Eubio) and incubated 16-18 hours at 37°C. Capillary-like structures within the Matrigel layer were then photographed and analyzed using Image J morphometry software.

### Apoptosis assay

5×10^4^ cells per well were cultured in a 24-well plate. After 24 hours, the cells were treated with 1 μM apelin-13. Apoptosis of cells was induced with an UV dose of 200mW/cm2 for 10 seconds. After 48 hours incubation, cells were fixed in 4% PFA for 10 min. Apoptosis assay was carried out using the “In Situ Cell Death Detection” kit (Roche, Mannheim, Germany) and the protocol recommended by the manufacturer. Finally, cells were coverslipped in Vectashield mounting medium containing DAPI (Vector Laboratories). Five random fields per slide were photographed and the percentage of TUNEL-positive cells among total cells was calculated and averaged.

### Western Blot Analysis

LECs exposed to 1 μM apelin in serum-free medium (EBM-2, Lonza) for different time durations were lysed in RIPA-buffer (Fischer Scientific, **Loughborough, UK)**, centrifuged, and protein was denatured before loading. Each sample was subjected to 12% sodium dodecyl sulfate (SDS)–polyacrylamide gel electrophoresis. The proteins were electro-transferred onto nitrocellulose membranes and immunodetected with antibodies against Akt-pAkt, Erk1/2-pErk1/2, S6-pS6 (all from Cell Signaling Technologies, Massachusetts, USA) and β-actin (Sigma). Blots were then incubated with appropriate HRP-labeled secondary antibodies (Thermo Fisher Scientific, Rockford, IL, USA) and signals were detected by using the ECL system (GE Healthcare, Dassel, Germany). Activation of signaling was quantified as the ratio of phosphorylated and total protein densitometry measurements.

### Matrigel plug assay

Groups of three C57BL/6 mice were injected subcutaneously with 0.25 ml Matrigel (BD Biosciences) containing PBS, 0.25 μg bFGF (Eubio), or 0.25 μg apelin (Phoenix Pharmaceuticals). One week after implantation, the plug was removed, and cryosections (5 μm) were prepared for immunohistochemistry. After labeling with rabbit anti-mouse LYVE-1 and rhodamine-conjugated goat anti-rabbit IgG (purchased from ReliaTech, Braunschweig, Germany and Jackson ImmunoResearch Inc., West Grove, PA, respectively), sections were examined using a Nikon Eclipse 80i microscope and digital images were captured using a SPOT digital camera (Diagnostic Instruments, Sterling Heights, MI). Quantitative analyses of the LYVE-1-positive lymphatic vessels in the Matrigels were performed using ImageJ software.

### *In vivo* tumor studies

Growth of the apelin-transfected B16 mouse melanoma cells was compared with that of control vector expressing cells in allograft tumors formed in 9-week-old female BDF1 ((C57BL/6 x DBA/2)F1) mice. According to the institutional animal welfare guidelines, all mice were maintained on a daily 12-h light/12-h dark cycle and were housed under pathogen-free conditions in microisolator cages with laboratory chow and water ad libitum. B16-apelin and B16-mock cells were grown to 80% confluence, harvested by trypsinization and washed twice. Tumor allografts were established by injecting mice subcutaneously with 1×10^6^ B16-apelin or B16-mock cells under anesthesia with a combination of tiletamine hypochloride, zolazepam hypochloride, xylazin and butorphanol. Tumor size was measured every two days with a caliper and expressed in mm^3^ by the formula for the volume of a prolate ellipsoid (length x width^2^ π/6), as described previously [15, 48]. Tumors were removed from mice after 18 days of growth and were fresh-frozen in liquid nitrogen for further analysis. Lymph vessel densities (LVDs) were determined by labeling of lymph capillaries with rabbit anti-mouse LYVE-1 (ReliaTech) and rhodamine-conjugated goat anti-rabbit IgG (Jackson ImmunoResearch). Three sections per tumor were analyzed using a Nikon Eclipse 80i microscope. Digital images were captured and analyzed with a SPOT digital camera (Diagnostic Instruments) and the ImageJ software, respectively and as described previously [49, 50].

To study the LN metastatic potential of B16-apelin primary tumors, in another set of *in vivo* tumor experiments, groups of ten mice were injected with B16-apelin or B16-mock cells (1×10^6^ cells per animal) in a total volume of 100 μl into the right hind footpad. Animals were euthanized and autopsied at 3 weeks postinoculation when the primary tumors reached 5-10 mm in diameter. Metastatic involvement of the ipsilateral inguinal sentinel LNs was evaluated with a stereomicroscope (Alpha, Woking, UK). The right-side inguinal LN of each animal were then collected, embedded in paraffin, serial sectioned, stained with haematoxylin and eosin and evaluated by a pathologist (BD).

### Statistical analysis

Continuous variables were compared with Student's t-test if the sample distribution was normal or with Mann-Whitney U test if the sample distribution was asymmetric. Categorical data were compared using Fisher's exact probability and χ^2^ tests. Differences were determined to be significant if p<0.05. All statistical analyses were done using GraphPad Prism 5.0 (GraphPad Software, Inc.; La Jolla, CA) software.
